# Effect of Partial Ba Substitutions on the Crystallization of Sr_2_TiSi_2_O_8_ (STS) Glass–Ceramics and on the Generation of a SAW Signal at High Temperatures

**DOI:** 10.3390/ma14164648

**Published:** 2021-08-18

**Authors:** Maurice Gonon, Florian Dupla, Hassan Alhousseini, Marc Duquennoy, Nikolay Smagin, Grégory Martic

**Affiliations:** 1 Materials Institute, University of Mons, Rue de l’Epargne 56, 7000 Mons, Belgium; florian-dupla@sfr.fr; 2 Institut d’Electronique de Microélectronique et de Nanotechnologie, Départment Opto-Acousto-Electronique (IEMN DOAE CNRS UMR 8520), Polytechnic University of Hauts-de-France, 59313 Valenciennes, France; Hassan.Alhousseini@etu.uphf.fr (H.A.); marc.duquennoy@uphf.fr (M.D.); Nikolay.Smagin@uphf.fr (N.S.); 3 Belgian Ceramic Research Center, Avenue Gouverneur Cornez 4, 7000 Mons, Belgium; g.martic@bcrc.be

**Keywords:** crystallization, fresnoite, glass–ceramics, high temperatures, piezoelectricity, preferential orientation, surface acoustic waves

## Abstract

Because of their characteristics, including a d_33_ of 10–15 pC/N and high stability up to temperatures over 1000 °C, polar glass–ceramics containing fresnoite crystals can be regarded as highly effective materials for applications requiring piezoelectricity at high temperatures. In the present paper we investigate barium substitutions in an Sr-fresnoite (STS) glass–ceramic. Two aspects are studied: first, the effect of the substitution on the preferential orientation of the crystallization, and second, the ability of the glass–ceramics to generate and propagate surface acoustic waves (SAW) at high temperatures. XRD analyses show that a 10 at.% substitution of Ba allows us to keep a strong preferential orientation of the (00l) planes of the fresnoite crystals down to more than 1 mm below the surfaces. Higher substitution levels (25 and 50 at.%), induce a non-oriented volume crystallization mechanism that competes with the surface mechanism. SAW devices were fabricated from glass–ceramic substrates with 0, 10 and 25 at.% Ba substitutions. Temperature testing reveals the high stability of the frequency and delay for all of these devices. The glass–ceramic with a 10 at.% Ba substitution gives the strongest amplitude of the SAW signal. This is attributed to the high (00l) preferential orientation and the absence of disoriented volume crystallization.

## 1. Introduction

In a few decades, piezoelectric materials have conquered many industrial sectors, whether in the military (hydrophone, depth sensors, security systems, etc.), the automobile (airbag sensors, injectors, etc.), the IT (hard-drive microactuators), the medical (ultrasonic therapy, insulin pumps, image acquisition, fetus heartbeat detection, etc.) or the commercial (resonators for radios/TV, lighters, alarm systems, music instruments, etc.) sector [1]. Several families of materials show piezoelectricity, but the most commonly encountered is that of the ferroelectric perovskite–type ceramics, such as lead titanate PbTiO_3_, barium titanate BaTiO_3_ and lead zirconate titanate Pb(Zr_x_Ti_1−x_)O_3_, also known as PZT [2,3].

In ferroelectrics, the crystals are divided in polar domains in which all the dipole moments show the same orientation. The equilibrium between these domains makes it so that the crystal itself shows no net polarization. However, by applying a strong external electrical field, the equilibrium is broken to the benefit of the domains showing the closest alignment to the direction of the field, resulting in a remanent polarization. Ferroelectrics exhibit high piezoelectric performances due to the large contribution of the motion of the domain walls to the electromechanical response. However, they undergo ageing by depolarization over time, resulting in the degradation of their piezoelectric characteristics [4]. Moreover, the rate of depolarization accelerates with the increase in temperature. It is usually admitted that the maximum service temperature of a ferroelectric is about one-half of its Curie temperature [5].

**Figure 1:**
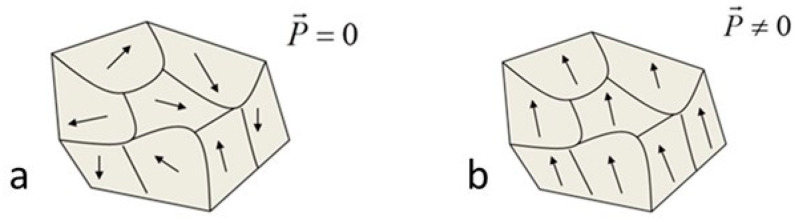
Polar moments in a non-ferroelectric pyroelectric polycrystal: (**a**) randomly oriented and (**b**) preferentially oriented.

In the pyroelectric group, very few compounds, such as fresnoite Ba_2_TiSi_2_O_8_, are non-ferroelectric. For those compounds, crystals are not divided into polar domains. The orientation of the dipole moments is uniform over the whole crystal, leading to a spontaneous polarization. However, in the case of a polycrystalline material, a random orientation of the grain makes null the overall polarization (Figure 1a). This means that non-ferroelectric pyroelectrics can only be used in the form of single crystals or preferentially oriented polycrystalline materials (Figure 1b). Due to the absence of the contribution of the motion of the domain walls, piezoelectric properties of these compounds are significantly lower than those of ferroelectrics. On the other hand, with polarization being structurally induced, their piezoelectric properties are highly stable over time, and they depolarize in temperature only if a pyroelectric/non-pyroelectric phase transition occurs, or at melting.

Fresnoite, Ba_2_TiSi_2_O_8_, is a mineral that was discovered in California in 1965; it received its name from the County of Fresno, where it was first extracted [6]. Two years later, the first crystallographic studies showed that the crystal structure of fresnoite leads to piezoelectric properties [7]. Fresnoite single crystals were synthesized shortly after their discovery. In 1973, Ba_2_TiGe_2_O_8_ single crystals were also synthesized and showed a similar structure and piezoelectric properties [8]. Since then, it has been demonstrated that different fresnoite-type compounds of formula A_2_TiB_2_O_8_ (A = Ba, Sr; B = Si, Ge) show piezoelectric properties. The most studied are Ba_2_TiSi_2_O_8_ (BTS), Sr_2_TiSi_2_O_8_ (STS) and Ba_2_TiGe_2_O_8_ (BTG). BTS and STS have quite the same crystal structure and belong to the P4bm space group [9,10,11]. They both crystallize in the tetragonal system, and their lattice parameters are a = 852.91 pm and c = 521.10 pm for BTS and a = 832.18 pm and c = 502.92 pm for STS. On its side, BTG is orthorhombic at room temperature, becomes tetragonal around 850 °C and also has a phase transition at around −50 °C [12]. The crystal structure of fresnoite is very particular because the titanium is at the center of a square-based pyramid, leading to a coordination number of 5 [10]. This characteristic is the source of permanent electrical dipoles in the c direction. Fresnoite is then pyroelectric, but, as previously said, it is not structured in ferroelectric domains. Consequently, macroscopic piezoelectricity can only be observed in a single crystal or in a polycrystalline material if crystals show a preferential orientation of the c direction.

Unlike the traditional ceramic technology based on the powder process, the glass–ceramic route can lead to highly oriented microstructures. A glass–ceramic is a polycrystalline material obtained by controlled crystallization of a glass precursor (called “parent” glass in this paper). Preferential orientation of the crystal domains can be observed in the material when the crystallization results from a surface mechanism [13,14]. However, the competition between surface and volume crystallization is a complex phenomenon that depends on many parameters, such as the glass composition, surface state and thermal-treatment parameters. In the early 1980s, Halliyal et al. developed numerous polar glass–ceramics based on non-ferroelectric pyroelectric compounds [15,16,17,18]. Among these glass–ceramics, some show fresnoite crystals. Since then, numerous works have been published and are detailed in the recent review of Wolfgang Wisniewski et al. [19].

Considering their characteristics, d_33_ of 10–15 pC/N and high stability up to temperatures over 1000 °C [19], polar fresnoite glass–ceramics are very good alternative materials for high temperature piezoelectric devices. Indeed, concurrent materials as quartz (SiO_2_), lithium nobiate LiNbO_3_, gallium phosphate GaPO_4_, or langasite (LGS) La_3_Ga_5_SiO_14_, must be used as single crystals and exhibit either quite low piezoelectric coefficients or a limited maximal work temperature [20,21,22,23,24,25,26]. Some new compounds are topics of research. This is the case of the interesting Aurivillius family [27,28,29] with Curie temperatures ranging from 750 to 950 °C and piezoelectric constants d_33_ lying between 6 and 18.5 pC/N. However, their manufacturing requires specific sintering methods, such as hot-pressing or SPS (Spark Plasma Sintering).

In their paper, Davis et al. [30] highlight the potential of fresnoite glass–ceramics for applications, such as accelerometers for turbine engines or pressure sensors. In a previous paper [31], we demonstrated the ability of an STS glass–ceramic to generate and propagate SAW up to a temperature of 900 °C.

The crystallization treatment is the most important step of the synthesis of a fresnoite glass–ceramic because it determines the final piezoelectric properties. As explained by Gerace et al. [32], oriented crystal growth can be achieved by using kinetic and thermodynamic controls. The kinetic control is the result of the anisotropic growth rate of the crystal. Together with a surface nucleation mechanism, the kinetic selection can induce a preferential orientation of the fastest-growing crystallographic direction perpendicular to the surface of the sample [19]. On its side, the thermodynamic route involves an external stimulation, such as an electrical field, which favors the orientation of the nascent crystals in the glass.

From the composition point of view, a silica excess with respect to the fresnoite stoichiometry is needed to successfully melt a homogeneous glass [33]. Moreover, a strong volume crystallization occurs during the devitrification treatment if the silica excess is not sufficient. Different techniques have been tested to enhance the preferential orientation of the fresnoite crystals: thermal gradient [16,34], electrochemical nucleation [35,36] and ultrasonic surface treatment with crystalline fresnoite particles [37]. However, the most versatile and convenient in terms of equipment consists in finding the ideal combination of composition/isothermal treatment to avoid volume crystallization. Nevertheless, the settings (heating rate, maximal temperature, duration and environment) are difficult to define and strongly dependent on the glass composition.

In the case of STS glass–ceramics, Patschger et al. [38] have highlighted the change in orientation of planes with the depth when crystallizing a glass 2SrO 1TiO_2_ 2.75SiO_2_ (reference ST 0.75S according to nomenclature proposed in Reference [19]). They covered the parent glass surface with alumina and graphite powder before the crystallization treatment, and the orientations and piezoelectric coefficients were compared to a non-covered sample. After crystallization, the glass–ceramics show a preferential orientation of the (002) fresnoite plans parallel to the surfaces, but a (201) orientation appears in a depth of 50 µm for the sample covered with graphite, and 200 µm for the alumina-covered sample. They measured the d_33_ after a 500 µm polishing and obtained a d_33_ = 16.5 pC/N with the graphite, d_33_ = 14.9 pC/N with the alumina, and d_33_ = 10.8 pC/N for the reference sample. These results show that the piezoelectric coefficient of the (201) oriented fresnoite is high and is dependent on the initial (002) orientation at the surface.

In their work, Maury et al. [39] investigated the synthesis STS glass–ceramics from parent glasses in the SrO–TiO_2_–SiO_2_–K_2_O–B_2_O_3_ system. Here also, a preferential orientation of the (002) plans at the surface was observed with all compositions after a single isothermal heat treatment at 900 °C, but only glasses with a low content of K_2_O kept this preferential orientation in the bulk down to 2 mm deep.

On basis of this latter work, Renoirt et al. [40] investigated the crystallization of STS from parent glasses belonging to the SrO–TiO_2_–SiO_2_–Al_2_O_3_–K2O system. The composition 2SrO 1TiO_2_ 3.3SiO_2_ 0.2K_2_O 0.1Al_2_O_3_ (reference ST 1.3S + 0.2K_2_O + 0.1Al_2_O_3_), crystallized at 900 °C, shows the best crystal orientation, but replicability tests highlight that the (002) preferential orientation observed at the surface of most of the time changes to (201) after a depth of about 300 µm. Despite this, the charge coefficient d_33_ reaches 11 to 12 pC/N and is not significantly influenced by the preferential orientation (002) or (201). In addition, High temperature XRD shows the stability of the STS phase in this glass–ceramic up to 1000 °C. It is this composition that we recently used to demonstrate the possibility to realize and operate a SAW device at a high temperature [31]. Despite significant variations in the output signal amplitude with temperature, it remains readable up to 900 °C. In addition, the device showed quite good stability of its frequency and sound velocity with temperature, which is a prerequisite for the design of a sensor.

In the work hereafter presented, we study first the influence of a partial substitution of Ba to Sr on the crystallization of the glass–ceramic composition ST 1.3S + 0.2K_2_O + 0.1Al_2_O_3_ and on the preferential orientation of the (002) plan in the bulk of the material. We then compare the ability of non-substituted and Ba-substituted glass–ceramics to operate a SAW device at high temperature.

## 2. Materials and Methods

### 2.1. Synthesis of the Glass–Ceramics

Parent glasses were prepared according to the procedure presented in Reference [40]. Reagent grade SrCO_3_ or BaCO_3_ (Alfa Aesar, Karlsruhe, Germany, 99.99%), TiO_2_ (VWR, Fontenay sous Bois, France, 99.99%), SiO_2_ (Sigrano, Maastricht, The Netherlands, 97–99.98%), Al_2_O_3_ (Almatis, Ludwigshafen, Germany, +99%) and K_2_CO_3_ (VWR, Fontenay sous Bois, France, +99%) were wet-mixed in isopropanol. After complete evaporation of the isopropanol, the melting of the powder mix was realized in a Pt/Au 95/5 crucible at 1500 °C for 2 h, thanks to a furnace Nabertherm HT 16/18. The melt was cast in 70 × 70 × 6 mm plates, in a steel mold. To release internal stresses and avoid cracks, these plates were annealed for 2 h at 700 °C, before slow cooling inside the turned off furnace. Each glass plate was surface polished (grade 220) and cut in parallelepipeds of 30 × 30 × 5 mm for the crystallization tests and 60 × 25 × 5 mm for the SAW devices.

The four compositions investigated are presented in Table 1. The composition Ba(0) corresponds to ST 1.3S + 0.2K_2_O + 0.1Al_2_O_3_ previously investigated [31,40]. In the compositions Ba(10), Ba(25) and Ba(50), 10 at.%, 25 at.% and 50 at.% of barium are respectively substituted to the strontium.

The crystallization treatment was performed in a furnace Nabertherm LT 40/12. Sample are placed in an alumina powder bed (Almatis CT 3000, Ludwigshafen, Germany) to ensure a good homogeneity of the temperature. Based on the previous works, the following temperature schedule was applied:

- 300 °C/h from room temperature to 900 °C;
- Dwell time of 20 h at 900 °C;
- Slow cooling in switch-off furnace;
- When needed, shorter dwell times are applied.

### 2.2. Investigation of the Crystallization

**Figure 2:**
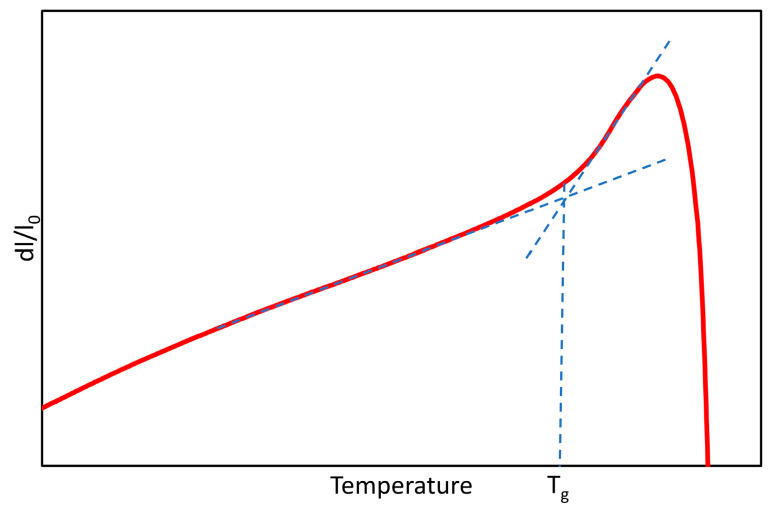
Principle of measurement of the glass-transition temperature from the dilatometry curve of a glass.

Glass-transition temperatures (T_g_) of parent glasses and residual glasses in the final glass–ceramics are obtained by dilatometry analyses performed on a horizontal dilatometer NETZCH DIL 402 C with a heating rate of 10 °C/min. Figure 2 shows the principle of measurement of T_g_ from the slope break of the curve. The accuracy of the measurement is about ±2 °C.

Parent glasses and glass–ceramics densities were calculated by using the Archimedes method in water, with specimens of about 10 g, and using a 0.001 accurate scale. Each measurement is repeated three times on three specimens of the same type. The precision of the measurement is about ±0.01 g/cm^3^.

Crystalline-phase analyses were carried out by XRD with a Siemens D5000 θ-2θ diffractometer (BRUKER, Karlsruhe, Germany), using a Co K_α_ radiation source (λ_K__α1_ = 178.897 pm) and Ni filter. To characterize the evolution of the orientation of the crystals over depth, the main surfaces of the samples were analyzed as obtained after crystallization and after successive grinding steps. Powder-milled samples were also analyzed to control the effectiveness of the Ba substitution by the measurement of the unit cell parameters. This measurement was realized by means of the Burker EVA software (BRUKER, Karlsruhe, Germany) and the function “tune cell” that allows small changes in the parameters of the unit cell given by the JCPDS cards. Accuracy of the method is ±1 pm and was determined by the measurement of the unit cell parameters of an alumina powder (Almatis CT 3000, Ludwigshafen, Germany) with respect to the JCPDS card # 00-010-0173. In addition, the same alumina powder was added to the glass–ceramic powders as a reference for the 2 scale.

### 2.3. SAW Testing Devices

**Figure 3:**
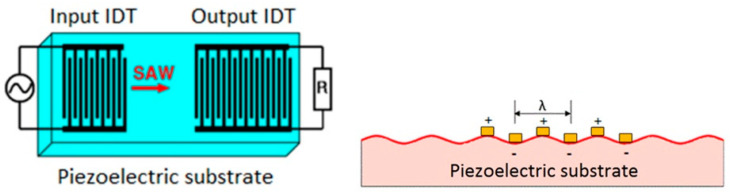
Configuration of a two ITDs SAW device.

A surface acoustic wave device is generally composed of two interdigitated transducers (IDT) deposited on a piezoelectric substrate (Figure 3). The input IDT, also called emitter, converts a sinusoidal electrical signal in a mechanical surface wave. This acoustic wave propagates at the surface of the substrate until reaching the output IDT, also called the receiver. This electrical/mechanical conversion is used for various purposes in SAW systems, such as sensors, selective filters and delay lines [41,42,43].

**Figure 4:**
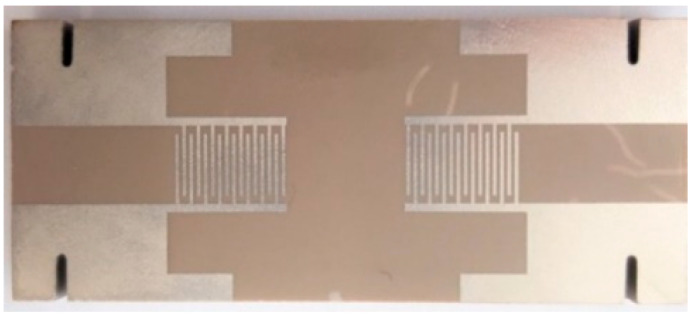
Glass–ceramics SAW device.

The surface acoustic wave device used for the tests was realized on a 60 × 25 × 5 mm glass–ceramic substrate (Figure 4). The IDTs were realized by a two-step metal-coating by sputtering and laser ablation, as it is detailed in Reference [31]. A 40 nm–thick adhesion layer of constantan (55 wt.% Cu, 45 wt.% Ni) is deposited prior to the 200 nm–thick platinum conductive layer. Sputtering is realized thanks to a BAL-TEC MED 020 equipment (Leica Microsystems, Wetzlar, Germany) working under an argon atmosphere. The targeted frequency for the IDTs is 2 MHz, the same as that of the SAW device successfully tested in Reference [31].

An Agilent 3320a arbitrary waveform generator was used to send the sinusoidal signal to the input IDT. Electrical connections to the input and output IDTs were realized by using Kanthal^®^ A-1 wires (KANTHAL AB, Hallstahammar, Sweden) attached to notches, and contact was ensured by silver conductive paste. The output signal was observed on an oscilloscope, and a MATLAB (Matlab 2017b, MathWorks, Natick, MA, USA) program was used to record the signal. The SAW device was placed inside a tubular furnace. A heating ramp of 5 °C/minutes was used. The output signal was recorded for every 10 °C increase in heating.

## 3. Results

### 3.1. Densities and Glass Transition Temperatures

**Figure 5:**
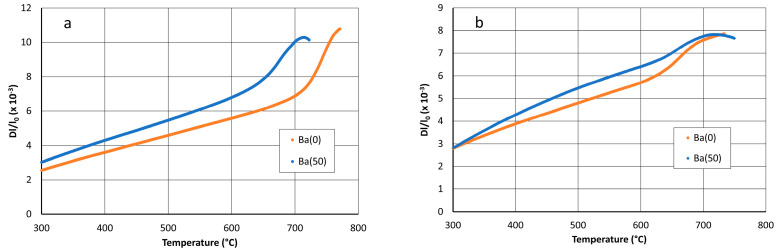
Dilatometry curves of Ba(0) and Ba(50) parent glasses (**a**) and glass–ceramics (**b**).

The glass transition temperature and density of the parent glasses and glass–ceramics are given in Table 2. The Ba substitution significantly lowers the glass-transition temperature of the parent glass that decreases from 712 to 640 °C with a 50% substitution (Figure 5a). This shows that Ba^2+^ cations behave as a stronger fluxing agent than the Sr^2+^ in the glass. On the contrary, although some differences in the dilatometric curves of the glass–ceramics are visible (Figure 5b), the T_g_ remains in the range 630–635 °C (Table 2). This suggests that the composition of the residual glass is not significantly modified, as it was expected.

**Figure 6:**
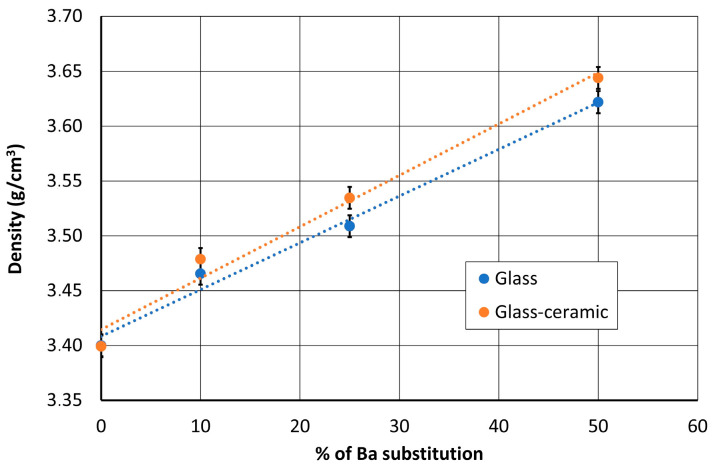
Evolution of the densities of the parent glasses and glass–ceramics with the Ba substitution.

Figure 6 shows that the density of the glass–ceramic increases nearly linearly with Ba substitution. This agrees with the higher density of BTS (4.439 g/cm^3^) than STS (3.884 g/cm^3^). The Ba substitution also slightly increases density variation during the crystallization. The crystallization of the non-substituted composition induces no volume change. For the 50% substituted, the increase in density from 3.62 to 3.64 g/cm^3^ leads to a small volume shrinkage of about 0.8%.

### 3.2. Crystallization

#### 3.2.1. Effect of the Ba Substitution on the Characteristics of the Fresnoite Crystals in the Glass–Ceramic

**Figure 7:**
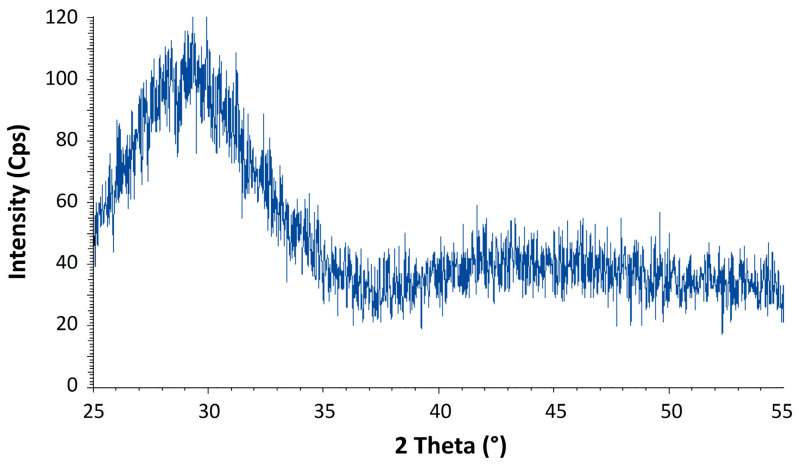
Diffraction pattern of the parent glass Ba(50).

The XRD patterns collected on the parent glass that were obtained after melting confirm the amorphous state of the materials, as shown in Figure 7 for the composition Ba(50).

**Figure 8:**
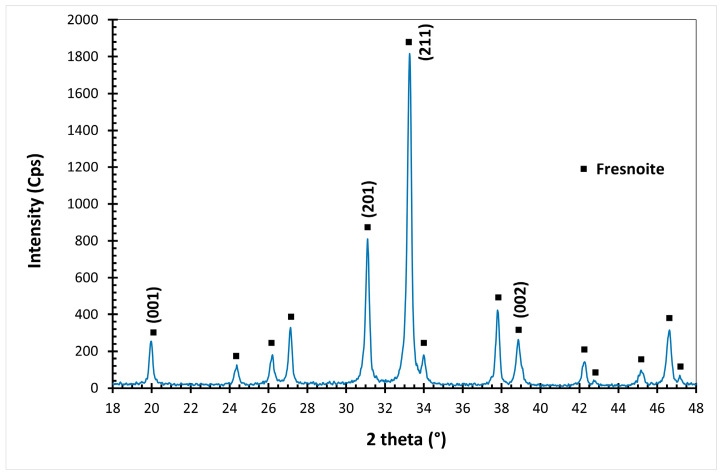
Diffraction pattern of a powder-milled glass–ceramic Ba(50).

**Figure 9:**
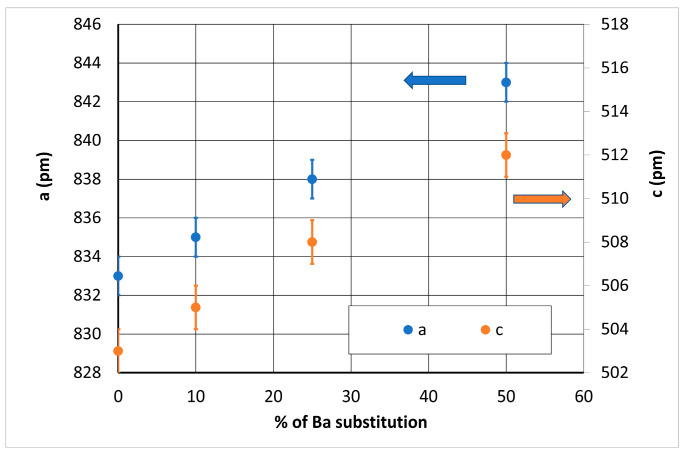
Evolution of the unit cell parameters with the percentage of Ba substitution.

After the crystallization treatment, XRD analyses realized on the powder-milled samples exhibits only the diffraction peaks corresponding to Fresnoite (Figure 8). The unit cell parameters measured on all the compositions are given in Table 3. As expected, the parameters increase with the Ba substitution (Figure 9) and range between those of STS, a = 832.180 pm and c = 502.920 pm (JCPDS # 00-039-0228, and BTS, a = 852.910 pm and c = 521.100 pm (JCPDS # 00-022-0513).

#### 3.2.2. Effect of the Substitution on the Orientation of the Fresnoite Crystals

As previously mentioned in the Introduction section, the crystallization STS glass–ceramics by isothermal treatments leads to (002) preferential orientation of the fresnoite plans at the surface, but a tilt to (201) plans may occur in depth. To investigate this possible evolution in the Ba substituted compositions, XRD analyses are realized after successive grinding steps.

**Figure 10:**
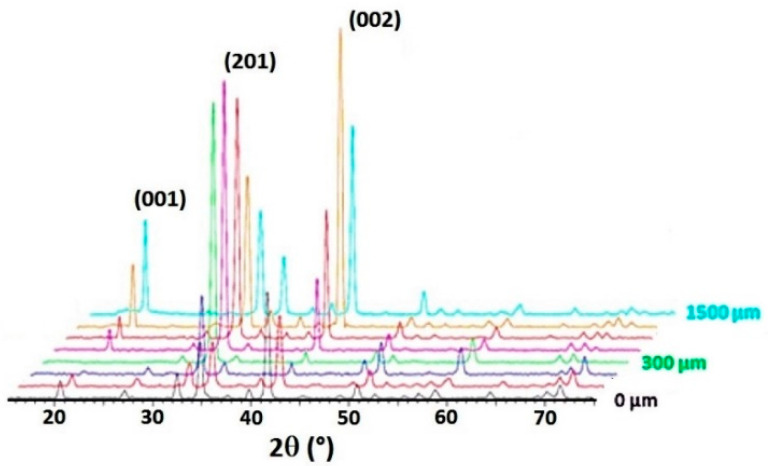
Evolution over depth of diffraction patterns for glass–ceramic Ba(0).

**Figure 11:**
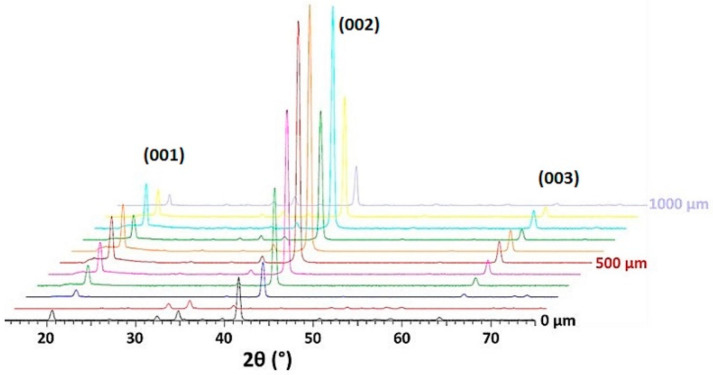
Evolution over depths of diffraction patterns of glass–ceramic Ba(10).

On the non-substituted Ba(0) composition, the XRD analyses show the presence of this tilt (Figure 10). The (002) plans give the strongest diffraction peak at the surface of the sample. In the bulk, the intensity of the diffraction peak (201) gradually increases until becoming the main peak at a depth of 300 µm. On the contrary, for glass–ceramic Ba(10), a strong intensity of the (00l) diffraction peaks is kept over the entire analyzed depth of 1000 µm (Figure 11).

To quantify the preferential orientation of the (002) and (201) plans, R factors based on the relative intensities of the (002), (201) and (211) diffraction peaks are calculated:

(1) $$R\left(201\;or\;002\right)=\frac{I\left(201\;or\;002\right)}{I\left(201\;or\;002\right)+I\left(211\right)}$$

The (211) diffraction peak is the strongest peak for a non-oriented sample. The R factor reaches 1 if the considered plans are strongly preferentially oriented. Conversely, according to the relative intensities given by the JCPDS cards of STS and BTS, the R factor for a non-oriented sample is around 0.3 for plans (201) and 0.18 for plans (002).

**Figure 12:**
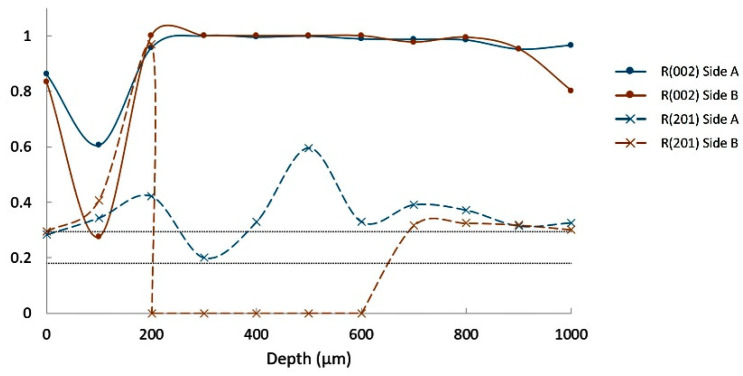
Orientation ratios R(002) and R(201) for composition Ba(10).

**Figure 13:**
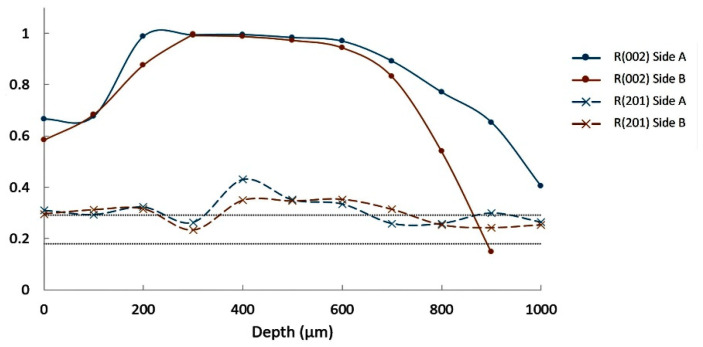
Orientation factors R(002) and R(201) for composition Ba(25).

**Figure 14:**
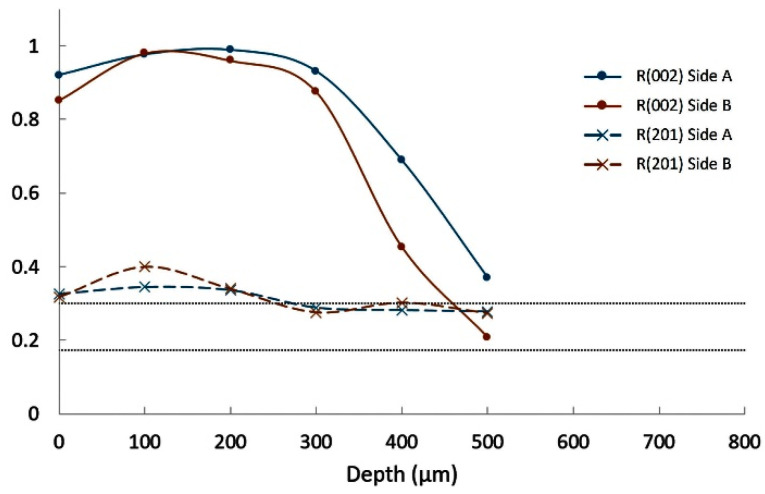
Orientation factors R(002) and R(201) for composition Ba(50).

Figure 12 shows the evolution of these R factors over depth from both sides (called side A and side B) of the Ba(10) glass–ceramic. After some variations over the first 200 µm, R(002) remains near 1 down to 1000 µm. This confirms the strong preferential orientation of the (002) plans. When increasing the Ba substitution to 25 at.%, strong values of R(002) are also observed but only down to 700–900 µm, as it is shown in Figure 13. This depth of high orientation is only 300–500 µm for composition Ba(50) (Figure 14).

**Figure 15:**
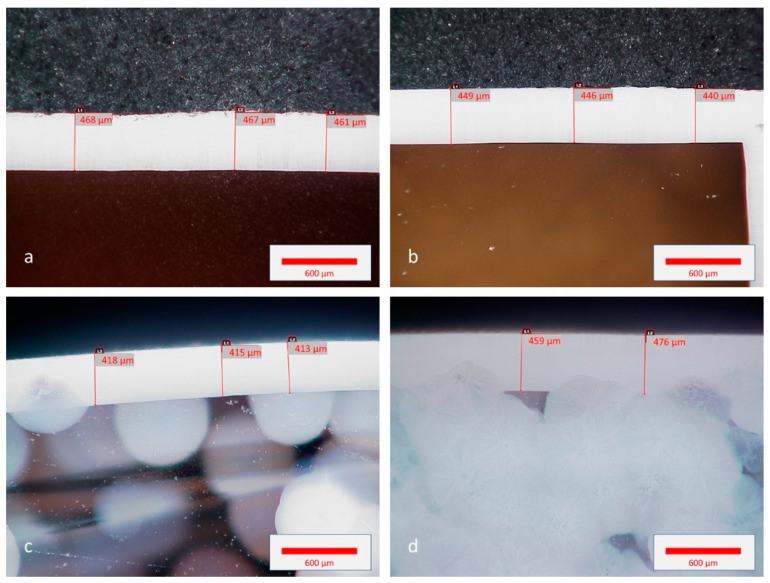
Optical microscope images of the cross-section of the glass–ceramics Ba(0) (**a**), Ba(10) (**b**), Ba(25) (**c**) and Ba(50) (**d**) after thermal treatment of 2 h at 900 °C.

Cross-section images of the glasses after a thermal treatment of 2 h at 900 °C show the crystallized layer that has propagated from the surface (Figure 15). They also show volume crystallization for compositions Ba(25) and Ba(50). The thickness of the surface crystallized layer is between 420 and 470 µm and does not seem to be related to the composition.

As a reminder, it was shown in Section 2.1. that the Ba substitution modifies the T_g_ of the parent glass but not that of the residual glass. This indicates that the surface crystallization rate is probably more influenced by characteristics of the residual glass, as it was highlighted in Reference [40], than those of the parent glass. Conversely, the clear increase in volume crystallization with the Ba content can be related to the change in the parent glass characteristics. Consequently, when increasing the Ba substitution, the competition between the volume and surface mechanisms lowers the thickness of the orientated crystalline layer.

### 3.3. Testing of the SAW Devices

Despite of the volume crystallization, the Ba substitution allows us to keep the (00l) orientation over the first millimeter below the surface. For this reason, two SAW devices were realized with the glass–ceramics Ba(10) and Ba(25). Those two devices are compared with a third one prepared from composition Ba(0) that shows a (201) preferential orientation. The true frequency of the IDTs, the delay between the input and output signal (i.e., the time of fly), and the amplitude of the output signal were recorded from RT up to 950 °C (limit due to the loss of signal due to the melting of the silver contacts).

**Figure 16:**
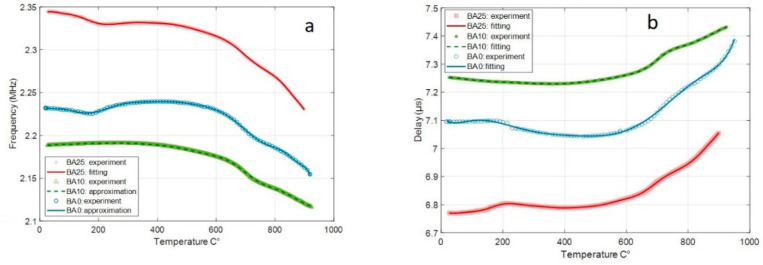
Evolution of the frequency (**a**) and delay (**b**) of the SAW devices.

Figure 16a,b respectively shows the evolution of the frequency and delay with temperature. Both are very stable from RT up to 600 °C for the three compositions. Next, between 600 and 900 °C, the frequency weakly decreases, whereas the delay increases. Those characteristics of the SAW devices are influenced by the evolution of the elastic properties of the substrate and its thermal expansion coefficient. This issue is discussed in Section 4.

**Figure 17:**
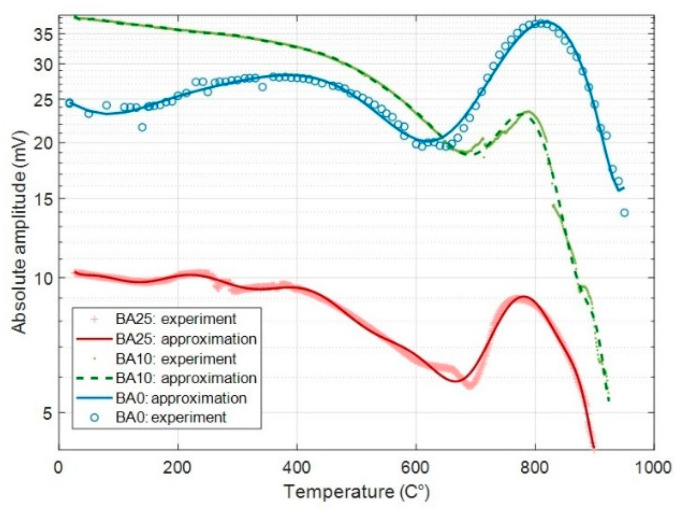
Evolution of the amplitude of the output signal with temperature.

Conversely, the amplitude of the output signal strongly varies with temperature (Figure 17). In addition, although the three devices qualitatively show similar behaviors, the amplitude for the composition Ba(25) is significantly lower. The amplitude of the electrical signal generated by the output ITD is a function of the effectiveness of the IDT in converting the SAW but also that of the substrate in propagating it. Therefore, its evolution with temperature depends both on that of the elastic properties and that of the piezoelectric properties of the substrate. This issue is also discussed in Section 4.

## 4. Discussion

### 4.1. Effect of the Substitution on the Characteristics of Glass–Ceramics

As noted in the introduction, the literature highlights that suitable Sr-fresnoite glass ceramic compositions exhibit a surface crystallization mechanism that favors a preferential orientation of the crystals by kinetic selection. At the surface, a preferential orientation of the (00l) plans is usually observed but is rarely kept in the bulk [44]. Wisniewski et al. explain the preferential orientation of the plans at the surface from the crystal structure and the diffusion rates of the atoms in the parent glass [45]. The lattice planes with the highest number of network formers per area are parallel to the surfaces. The change of the preferential orientation occurring in the bulk is related to the fastest growing crystallographic direction. Numerous works report that the preferential orientation of the (00l) plans disappears at the benefit of the (201) plans in the bulk [38].

**Figure 18:**
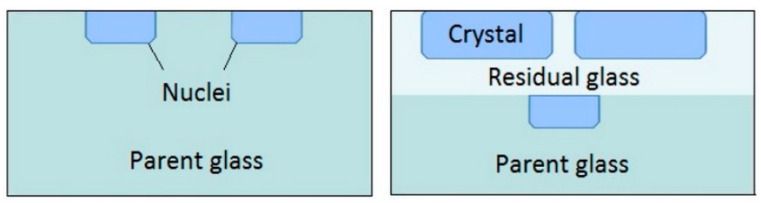
Supposed mechanism of surface crystallization by Maury et al. (adapted from [39]).

Maury et al. [39] assume that the nucleation and growth of the (00l) plans parallel to the free surfaces of the parent glass may be due to a significant anisotropy of the surface energy densities of the STS crystal. In depth, they explain that the propagation of the crystallization front is influenced by the properties of an interface parent glass/residual glass (Figure 18).

Renoirt et al. have investigated the effect of the composition of the residual glass on the crystallization of STS in the Sr–Ti–Si–K-Al–O System [40]. They conclude that the viscosity of the residual glass at the crystallization temperature influences the speed of the crystallization front and the preferential orientation: the higher the viscosity, the slower the speed and the better the orientation.

In the present work, the substitution of Ba in the composition ST 1.3S + 0.2K_2_O + 0.1Al_2_O_3_ lowers the glass-transition temperature of the parent glass, but not that of the residual glass (Table 2). Consequently, the viscosity of the parent glass at the dwell temperature of the thermal treatment (i.e., 900 °C) decreases. This does not influence the growth rate of the oriented surface layer (Figure 15a–d), which seems to be mainly governed by the properties of the residual glass. Conversely, this decrease in viscosity promotes the volume crystallization (Figure 15c–d), so that the thickness of the oriented crystalline layer falls well below 1 mm for 50 at.% substitution (Figure 14). On the other hand, for all the Ba substituted compositions investigated, the tilt from the (002) to the (201) preferential orientation does not appear (Figure 12, Figure 13 and Figure 14). Thus, this tilt seems not to be simply linked to the growth rate but is possibly influenced by the characteristics of the transition zone between the crystallized layer and the underlying parent glass.

### 4.2. Test of the SAW Devices

Glass–ceramic substrates with 0, 10 and 25 at.% Ba substitutions were used for the fabrication and test of SAW devices. The substitution has no significant effect on the frequency of the SAW signal and on its shift occurring with the increase in temperature (Figure 16a). The frequency is linked to the SAW velocity V_SAW_ and the spacing *p* of the IDTs:

(2) $$f_{0}\;=\;\frac{V_{\mathrm{SAW}}}{\lambda }\;=\;\frac{V_{\mathrm{SAW}}}{p}$$

The frequencies of the devices are close, indicating thereby no significant differences in SAW velocities. These are between 2760 and 2950 m/s at room temperature. They are only slightly higher than those given in the literature for fresnoite single crystals. Indeed, Sagnard et al. obtained a velocity of 2572 m/s for Ba_2_TiSi_2_O_8_ [46], and Ito et al. obtained 2640 m/s for Ba_1.2_Sr_0.8_TiSi_2_O_8_ and 2680m/s for Ba_2_TiSi_2_O_8_ [47].

When increasing temperature, the velocity V_SAW_ is influenced by the evolution of the elastic properties of the glass–ceramic, whereas the change in the spacing p is due to the expansion coefficient. The respective effects of these two parameters nearly offset each other below 600 °C. This is confirmed by the evolution of the delay with the temperature (Figure 16b), which is linked to the spacing D between the IDTs and the SAW velocity:

(3) $$t=\;\frac{D}{V_{\mathrm{SAW}}}$$

The delay shows a remarkable stability between RT and 600 °C for the three devices. This is a very interesting point for sensor applications, as the variation of the delay due to an external stimulation (e.g., pressure, environment, etc.) is often the working principle of SAW sensors.

Above 600 °C, the significant increase of the thermal expansion coefficient above the T_g_ (Figure 5b) is probably the main cause of the lowering of the frequency and the increase of the delay.

The evolution of the output signal amplitude with temperature (Figure 17) was discussed in Reference [31] for the unsubstituted composition Ba(0). The variations observed above the T_g_ of the residual glass were explained by the gradual softening of residual glass. In a first step, between 600 and 800 °C, the moderate softening makes it so that the fresnoite crystals are less constrained, which leads to an increase in the amplitude of the input signal, while the damping of the signal during propagation remains weak. Above 800 °C, the strong lowering of the viscosity of the residual glass induces a drastic increase in the damping. For temperatures below the T_g_ of the residual glass, the variation in amplitude was attributed to internal stress on the fresnoite crystal due to thermal expansion mismatch with the glass matrix.

In the present work, we qualitatively observe the similar evolution of the amplitude for all the tested devices. However, the amplitude at room temperature is higher for composition Ba(10) than for the composition Ba(0), with respectively 40 and 25 mV. This can be the result of the (002) preferential orientation instead of the (201) preferential orientation, which possibly increases the effectiveness of the ITDs. Nevertheless, the amplitude for composition Ba(25), that also shows a (002) preferential orientation, is much weaker (10 mV). This may be due to the volume crystallization that limits the depth of preferential orientation to the first 600–700 µm (Figure 13).

When increasing the temperature, the decrease in amplitude is much stronger for Ba than for the substituted compositions, with only 50% of the room-temperature value when the temperature reaches 650 °C, against 80% for the substituted composition. As previously said, the evolution of the SAW signal amplitude below T_g_ is presumably essentially due to the internal stress state induced by the thermal expansion mismatch. In Reference [31], we highlighted the possible occurrence of interfacial damages between the crystals and glass matrix. The effect of the mechanical strain on the piezoelectric response of fresnoite crystal must also be considered. At this stage of the work, detailed investigation has not yet been realized. To do so, an experimental device will be realized for characterizing separately the input and output signal amplitudes by means of a laser vibrometer. Anyway, the amplitude of the output signal remains rather at a high level, up to more than 800 °C, especially in the case of composition Ba(0) and Ba(10), and amplitude variations are not a critical problem for the application to the design of SAW sensor. Therefore, future work will focus on the fabrication of an operational system, such as a high temperature pressure sensor.

## Figures and Tables

**Table 1:** Compositions of the glass–ceramics.

Reference	SrO	BaO	TiO_2_	SiO_2_	K_2_O	Al_2_O_3_
Ba(0)	2.0	0.0	1	3.3	0.2	0.1
Ba(10)	1.8	0.2	1	3.3	0.2	0.1
Ba(25)	1.5	0.5	1	3.3	0.2	0.1
Ba(50)	1.0	1.0	1	3.3	0.2	0.1

**Table 2:** Densities and glass-transition temperature of parent glasses and glass–ceramics.

Composition	Parent Glass Density ±0.01 g/cm^3^	T_g_ of Parent Glass ±2 °C	Glass–Ceramic Density ±0.01 g/cm^3^	T_g_ of Glass–Ceramic ±2 °C
Ba(0)	3.40	712	3.40	634
Ba(10)	3.47	702	3.48	632
Ba(25)	3.51	681	3.53	630
Ba(50)	3.62	640	3.64	632

**Table 3:** Unit cell parameters of the fresnoite crystals in the different glass–ceramics.

Composition	A (pm) ± 1	C (pm) ± 1
Ba(0)	833	503
Ba(10)	835	505
Ba(25)	838	508
Ba(50)	843	512

## Data Availability

Not applicable.

## References

[1] Heywang W., Lubitz K., Wersing W. (2008). Piezoelectricity Evolution and Future of a Technology.

[2] Uchino K. (2010). Advanced Piezoelectric Materials: Science and Technology.

[3] Hemangi K., Deore H.A., Pranita P. (2008). Review on Advanced Piezoelectric Materials (BaTiO_3_, PZT). JETIR.

[4] Lupascu D.C., Genenko Y.A., Balke N. (2006). Aging in ferroelectrics. J. Am. Ceram.

[5] Turner R.C., Fuierer P.A., Newnham R.E., Shrout T.R. (1994). Materials for high-temperature acoustic and vibration sensors—A review. Appl. Acoust..

[6] Alfors J.T., Stinson M.C., Matthews R.A., Pabst A. (1965). Seven new barium minerals from Eastern Fresno County. Am. Min..

[7] Moore P.B., Louisnathan J. (1967). Fresnoite: Unusal titanium coordination. Science.

[8] Kimura M., Doi K., Nanamatsu S., Kawamura T. (1973). A new piezoelectric crystal: Ba_2_Ge_2_TiO_8_. Appl. Phys. Lett..

[9] Höche T., Rüssel C., Neumann W. (1999). Incommensurate modulations in Ba_2_TiSi_2_O_8_, Sr_2_TiSi_2_O_8_, and Ba_2_TiGe_2_O_8_. Solid State Commun..

[10] Moore P.B., Louisnathan J. (1969). The crystal structure of fresnoite, Ba(TiO)Si_2_O_7_. Z. Krist..

[11] Höche T., Neumann W., Esmaeilzadeh S., Uecker R., Lentzen M., Rüssel C. (2002). The crystal structure of Sr_2_TiSi_2_O_8_. J. Solid State Chem..

[12] Markgraf S.A., Bhalla A.S. (1989). Low-temperature phase transition in Ba_2_TiGe_2_O_8_. Phase Transit..

[13] Müller R., Zanotto E.D., Fokin W.M. (2000). Surface crystallization of silicates glasses: Nucleation and kinetics. J. Non. Cryt. Solids.

[14] Schmelzer J., Pascova R., Möller J., Gutzow I. (1993). Surface-induced devitrification of glasses: The influence of elastic strains. J. Non. Cryt. Solids.

[15] Halliyal A., Bhalla A.S., Newnham R.E. (1983). Polar glass ceramics—A new family of electroceramic materials: Tailoring the piezoelectric and pyroelectric properties. Mater. Res. Bull..

[16] Halliyal A., Safari A., Bhalla A.S., Newnham R.E., Cross L.E. (1984). Grain-oriented glass-ceramics for piezoelectric devices. J. Am. Ceram.

[17] Halliyal A., Bhalla S.A., Cross L.E., Newnham R.E. (1985). Dielectric, piezoelectric and pyroelectric properties of Sr_2_TiSi_2_O_8_ polar glass-ceramic: A new polar material. J. Mater. Sci..

[18] Halliyal A., Bhalla S.A., Newnham R.E., Cross E., Lewis M.H. (1989). Glass-ceramics for piezoelectric and pyroelectric devices. Glass and Glass-Ceramics.

[19] Wisniewski W., Thieme K., Rüssel C. (2018). Fresnoite glass-ceramics—A review. Prog. Mater. Sci..

[20] Bechmann R. (1958). Elastic and piezoelectric constants of alpha-quartz. Phys. Rev..

[21] Feifei C., Lingfeng K., Wei S., Chao J., Shiwei T., Fapeng Y., Lifeng Q., Chunlei W., Xian Z. (2019). The electromechanical features of LiNbO_3_ crystal for potential high temperature piezoelectric applications. J. Mater..

[22] Hauser R., Reindl L., Biniasch J. High-Temperature Stability of LiNbO_3_ Based SAW Devices. Proceedings of the IEEE Symposium on Ultrasonics.

[23] Fachberger R., Bruckner G., Knoll G., Hauser R., Biniasch J., Reindl L. (2004). Applicability of LiNbO_3_, Langasite and GaPO_4_ in high temperature SAW sensors operating at radio frequencies. IEEE Trans. Ultrason. Ferroelectr. Freq. Control.

[24] Nosek J., Pustka M. (2006). Determination of the electromechanical coupling factor of gallium orthophosphate (GaPO_4_) and its influence on resonance-frequency temperature dependencies. IEEE Trans. Ultrason. Ferroelectr. Freq. Control.

[25] Reiter C., Krempl M.W., Thanner H., Wallnöfer W., Worsch P. (2001). Material properties of GaPO_4_ and their relevance for applications. Ann. Chim. Sci. Matériaux.

[26] Takeda H., Tanaka S., Izukawa S., Shimizu H., Nishida T., Shiosaki T. Effective Substitution of Aluminum for Gallium in Langasite-Type Crystals for A Pressure Sensor Use at High Temperature. Proceedings of the IEEE Ultrasonics Symposium.

[27] Zhihang P., Dongxu Y., Qiang C., Deqiong X., Dan L., Dingquan X., Jianguo Z. (2014). Crystal structure, dielectric and piezoelectric properties of Ta/W codoped Bi_3_TiNbO_9_ Aurivillius phase ceramics. Curr. Appl. Phys..

[28] Wang Q., Wang C., Wang J., Zhang S. (2016). High performance aurivillius-type bismuth titanate niobate (Bi_3_TiNbO9) piezoelectric ceramics for high temperature applications. Ceram. Int..

[29] Bekhtin M.A., Bush A.A., Kamentsev K.E., Segalla A.G. (2016). Preparation and dielectric and piezoelectric properties of Bi_3_TiNbO_9_, Bi_2_CaNb_2_O_9_, and Bi_2.5_Na_0.5_Nb_2_O_9_ ceramics doped with various elements. Inorg. Mater..

[30] Davis M.J., Vullo P., Kocher M., Hovhannisyan M., Letz M. (2018). Piezoelectric glass-ceramic for high-temperature applications. J. Non. Cryst. Solids.

[31] Dupla F., Renoirt M.-S., Gonon M., Smagin N., Duquennoy M., Martic G., Erauw J.-P. (2020). A lead-free non-ferroelectric piezoelectric glass-ceramic for high temperature surface acoustic wave devices. J. Eur. Ceram. Soc..

[32] Gerace K.S., Mauro J.C., Randall C.A. (2021). Piezoelectric glass-ceramics: Crystal chemistry, orientation mechanisms, and emerging applications. J. Am. Ceram. Soc..

[33] Masai H., Tsuji S., Fujiwara T., Benino Y., Komatsu T. (2007). Structure and non-linear optical properties of BaO-TiO_2_-SiO_2_ glass containing Ba_2_TiSi_2_O_8_ crystal. J. Non Cryst. Solids.

[34] Ochi Y., Meguro T., Kakegawa K. (2006). Orientated crystallization of fresnoite glass-ceramics by using a thermal gradient. J. Eur. Ceram. Soc..

[35] Keding R., Rüssel C. (2000). Oriented glass-ceramic containing fresnoite prepared by electrochemical nucleation of a BaO-TiO_2_-SiO_2_-B_2_O_3_ melt. J. Non Cryst. Solids.

[36] Höche T., Keding R., Rüssel C. (1999). Microstructural characterization of grain-oriented glass ceramics in the system Ba_2_TiSi_2_O_8_. J. Mater. Sci..

[37] Ding Y., Masuda N., Miura Y., Osaka A. (1996). Preparation of polar oriented Sr_2_TiSi_2_O_8_ films by surface crystallization of glass and second harmonic generation. J. Non Cryst. Solids.

[38] Patschger M., Wisniewski W., Rüssel C. (2012). Piezoelectric glass-ceramics produced via oriented growth of Sr_2_TiSi_2_O_8_ fresnoite: Thermal annealing of surface modified quenched glasses. CrystEngComm.

[39] Maury N., Cambier F., Gonon M. (2011). Bulk crystallisation of (00l) oriented fresnoite Sr_2_TiSi_2_O_8_ in glass-ceramics of the Sr–Ti–Si–K–B–O system. J. Non Cryst. Solids.

[40] Renoirt M.-S., Maury N., Dupla F., Gonon M. (2019). Structure and Properties of Piezoelectric Strontium Fresnoite Glass-Ceramics Belonging to the Sr–Ti–Si–Al–K–O System. Ceramics.

[41] Tyagi S., Mahesh V.G. (2012). SAW and interdigital transducers. IJSER.

[42] Anghelescu A., Nedelcu M. New Piezoelectric Materials for SAW Filters. Proceedings of the SPIE, Advanced Topics in Optoelectronics, Microelectronics, and Nanotechnologies V.

[43] Schiopu P., Cristea I., Grosu N., Craciun A. Recent Developments in Surface Acoustic Wave Sensors. Proceedings of the SPIE, Advanced Topics in Optoelectronics, Microelectronics, and Nanotechnologies IV.

[44] Wisniewski W., Dimitrijevic J., Rüssel C. (2018). Oriented nucleation and crystal growth of Sr-fresnoite (Sr_2_TiSi_2_O_8_) in 2SrO·TiO_2_·2SiO_2_ glasses with additional SiO_2_. Cryst. Eng. Comm..

[45] Wisniewski W., Rüssel C. (2021). Oriented surface nucleation in inorganic glasses—A review. Prog. Mater. Sci..

[46] Sagnard M., Laroche T., Ballandras S. Surface Acoustic Waves Properties on Ba_2_TiSi_2_O_8_ for High Temperature Sensors. Proceedings of the International Ultrasonic Symposium (IUS).

[47] Ito I., Nagatsuma K., Ashida S. (1980). Surface acoustic wave characteristics of (Ba_2−x_ Sr_x_)TiSi_2_O_8_ crystals. Appl. Phys. Lett..

